# Dopamine Receptor D2 Gene (DRD2) Polymorphisms, Job Stress, and Their Interaction on Sleep Dysfunction

**DOI:** 10.3390/ijerph17218174

**Published:** 2020-11-05

**Authors:** Yu Jiang, Baoying Liu, Chuancheng Wu, Xiaoyan Gao, Yaoqin Lu, Yulong Lian, Jiwen Liu

**Affiliations:** 1 Department of Preventive Medicine, Fujian Provincial Key Laboratory of Environment Factors and Cancer, Key Laboratory of Environment and Health, School of Public Health, Fujian Medical University, Fuzhou 350012, China; jiangyu@fjmu.edu.cn (Y.J.); lby@mail.fjmu.edu.cn (B.L.); wcc@mail.fjmu.edu.cn (C.W.); 2 Department of Occupational and Environmental Health, School of Public Health, Xinjiang Medical University, Urumqi 830001, China; 15199142607@163.com (X.G.); lyq_superior@163.com (Y.L.); 3 Department of Occupational and Environmental Health, School of Public Health, Nantong University, Nantong 226019, China

**Keywords:** job stress, dopamine receptor D2 (DRD2), sleep dysfunction, gene-environment interaction, sDA

## Abstract

Recent studies have shown that incessant job stress could eventually result in sleep dysfunction (SD), and most importantly, the essential role dopamine receptor D2 (DRD2) gene polymorphisms play in the psychopathological mechanism of SD. The Effort-Reward Imbalance scale and the Pittsburgh Sleep Quality Index were both used to access SD and job stress (JS). A significant negative correlation was observed between the sDA levels and SD subscale scores (sleep efficiency, daytime dysfunction). The findings revealed that high levels of JS were linked to a higher SD score (OR = 2.13, 95% CI: 1.46–3.12). Likewise, the homozygous A1A1 genotype of DRD2 rs1800497 was more likely to be associated with SD (OR = 2.90, 95% CI: 1.75–4.82). Compared to participants with low JS and heterozygous A1A2/A2A2 genotype, those with both high JS and homozygous A1A1 genotype had a higher SD score (OR = 5.40, 95% CI: 2.89–10.11). The A1 allele of the DRD2 rs1800497 polymorphism also enhances the likelihood of SD when undergoing JS. Besides, subjects with low JS and the homozygous A1A1 genotype also showed an increased possibility for sleep dysfunction (OR = 2.05, 95% CI: 1.03–4.11). Our results suggest that the DA system may interrelate with JS to affect sleep.

## 1. Introduction

Sleep is a necessary bodily function for living. It is pivotal for both cognition function and the maintenance of robust brain functions, as well as the promotion of memory formation, learning, emotional well-being, neuronal activity, and a series of abilities [1,2]. Several studies have shown that long-term sleep disorders (SD) (such as deprivation, latency, nightmares, efficiency, and insomnia) have grave health repercussions. SD is associated with a host of chronic diseases such as depression, metabolic syndrome, cardiovascular diseases, or cancer [3,4,5,6]. Recent reports indicate that the prevalence of SD worldwide is on the rise. Notably, SD in the United States, England, and China account for between 20 and 40% of the cases worldwide [7,8,9].

Moreover, despite the health consequences associated with SD, the incidence of sick leave requests for professionals and the risk of occupational injuries have also increased [10,11]. These technical challenges have reduced general productivity [10]. Kessler and colleagues [8] provided an estimation of the effects of insomnia and work performance, in which annual insomnia-related costs range between $15 billion and $92 billion.

Workers often relate sleep deprivation to work-related factors and job stress (JS). Workers cite high work demands, bullying, and unfavorable work schedules to be among the factors causing SD. Nonetheless, organizational factors, effort-reward imbalance, and exposure to physical loading or chemical factors also contribute to sleep deprivation [12,13]. Besides, JS is the most prevalent self-reported cause of SD for the working class. JS disrupts sleep processes and circadian rhythms, as well as fatigue and insomnia, which results from improved cognition function and physical arousal [14,15].

A recent 5-year follow-up cohort study that evaluated the effort-reward imbalance and sleep disturbances showed that in male subjects, the effort-reward imbalance is a risk factor for the progression and development of SD. On the other hand, the association between effort-reward imbalances and SD in female subjects was only reflected in cross-sectional studies. The study recommended that improving social and psychological working conditions may reduce the chances of sleep disorders [16].

Whereas many studies have reported various risk factors for sleep dysfunction, data on the underlying causes are still scant. The degree to which the interaction between genetic and environmental factors influences the manifestation of sleep dysfunction is mostly undocumented. However, the length to which the associative relationship between environmental and genetic factors influences the manifestation of sleep dysfunction is still undocumented.

Nevertheless, some studies have shown the relationship between genetic factors and sleep dysfunction, sleep latency (SL), daytime dysfunction (DD), sleep efficiency (SE), and prolonged sleep time (PST) [17]. Over the past ten years, the influence of genetic factors on sleep quality (SQ) has also been defined. In their quest to define this relationship, most researchers have exploited epidemiological investigations of twin studies, pedigree studies, and parasitology studies [18,19]. Dopamine (DA) is a crucial neurotransmitter in the CNS. DA is involved in several psychological and behavioral processes, such as attention, behavior, and motivation, which are all dependent on wakefulness [20].

Recent evidence shows dorsal raphe nucleus (DRN), substantia nigra pars compacta (SNc), and the ventral tegmental area (VTA), to be pivotal in the initiation and maintenance of sleep-wake cycles [21,22]. DA binds to different subtypes of receptors, including the D2 receptor. DRD2 is highly expressed in the VTA and the SNc that regulate the sleep-wake rhythm. It has been demonstrated that the genetic deletion of DRD2 genes in animals can lead to a significant reduction in wakefulness, accompanied by increased slow-wave sleep and rapid eye movement [23]. Importantly, the derived C allele is related to a shorter sleep duration [24]. The association between DRD2 gene polymorphisms and sleep disorders has been evaluated extensively in PD patients [25]. For example, a previous study showed a significant association between the DRD2 gene polymorphism Taq IA and the sudden onset of sleep in PD [26]. Additionally, the SNP rs1800497 (Taq1A) with an A1 allele is also related to the dysregulation of the body’s primary stress systems referred to as the hypothalamic-pituitary-adrenal (HPA) axis [27]. The HPA axis, one of the main axes of the neuroendocrine stress response, is hypothesized as a pathway by which job stress might be related to adverse health outcomes [28]. Research has shown that JS can activate the HPA axis and perturb normal sleep [29].

Given the close association between JS, SD, and the potential role genetics play in sleep disorders, it is timely to elucidate the molecular markers that define the relationship. The possible role of DRD2 SNPs in sleep development and the association of the gene’s interaction with JS (gene × stress interactions; G × E) on SD is worth interrogation. For the first time, this study will report the relationship between the three and help develop molecular markers for work-related sleep dysfunction in China.

## 2. Method and Materials

### 2.1. Study Participants

A total of 812 professionals in this cross-sectional were recruited from a public institution in Xinjiang province. The exclusion criteria were participants stationed abroad, age (<20 or >60 years of age), participants with chronic diseases, participants who were reported absent from work due to occupational injuries, and participants taking medication three months before the study. After the rigorous selection criteria, a total of 756 subjects were deemed eligible. However, a total of sixty-three participants voluntarily dropped out of the study. Ultimately, a total of 693 workers were included in the study. The ethical review committee (Xinjiang Medical University (2015006)) approved the study. All the participants received written details of the study and signed informed consent forms.

### 2.2. Clinical Measures

#### 2.2.1. SD and Grouping

The Pittsburgh Sleep Quality Index (PSQI) is a nineteen item self-rated questionnaire designed for measuring sleep disturbances and quality in clinical populations for one month [30,31]. A recent meta-analysis and systematic review showed the efficiency of PSQI in understanding SD as either a continuous or dichotomous construct [32]. The PSQI consists of seven clinically derived components: sleep disturbance, subjective SQ, SL, PSD, SE, DD, and sleep medications. Each domain ranges from zero to three. Individual scores were summed to obtain a global score ranging from zero to twenty-one, with higher global scores indicating poor SQ. Participants with a global score higher than five were classified as suffering from sleep dysfunction. In our study, the Cronbach’s alpha was 0.874, and all the seven subscales recorded values greater than 0.8.

#### 2.2.2. Job Stress (JS)

JS was calculated using the questionnaires based on the effort-reward imbalance (ERI) [33]. It is believed that job stress stems from an imbalance between work-related effort and an individual’s reward [33]. The twenty-three item Chinese version of the ERI questionnaire was adopted in the study, and they consisted of three dimensions: job effort (6 items), job reward (11), and over-commitment (6) [34]. A ratio of ERI was calculated by the formula:

sum effort score/sum reward score × c(1)

where ‘c’ is a correction factor weighting the different numbers of items in effort and effort (6/11) [34]. The ERI ratio of >1.0 shows an imbalance between effort and reward, that is JS [34]. On the other hand, effort, reward, and over-commitment were categorized using a median split. The median split distinguishes between low and high levels.

#### 2.2.3. Blood Sampling and Serum DA Measurements

A 5 mL fasting venous blood sample was collected from each participant between 7 a.m. and 9 a.m. at the workplace. Samples were centrifuged and stored at −80 °C until testing. Serum DA levels were measured by sandwich enzyme-linked immunosorbent assay using a commercially available kit (R&D Systems, Minneapolis, MN, USA). All blood samples were assayed by a research assistant blind to the clinical situation of the subjects.

#### 2.2.4. Genotyping

Genomic DNA was isolated and purified from the samples using a whole blood genome extraction kit. Genotypic screening for DRD2 rs1799732 and DRD2 rs1800497 for polymorphisms was carried out using the SNaPshot SNP assay (Table 1).

#### 2.2.5. Confounding Factors

Several occupational, socioeconomic, demographic, and lifestyle factors have been shown to be related to sleep. The following variables were considered in the study: Age, sex, level of education (junior college, bachelor’s, graduate and post-graduate), and marital status (married, unmarried, and divorced/widowed). Job tenure was classified under occupational factors. Besides, alcohol consumption, no (rarely/never) or yes (often/daily) and smoking, no (rarely/never) or yes (occasionally/daily), were considered as potential lifestyle factors that might affect the outcome.

### 2.3. Statistical Analysis

Statistical analyses were carried out using IBM SPSS version 20.0 (SPSS Inc., Chicago, IL, USA). Data were summarized using frequencies for categorical data. Chi-square tests were used to compare demographic characteristics. Partial correlation analysis was used to assess the correlations between JS, sDA, and sleep dysfunction. Odds ratios (ORs) and 95% confidence intervals (CIs) were determined for the associated with sleep dysfunction using logistic regression. For multiplicative interactions, we calculated *p*-values using the cross-product terms of two factors investigated in the logistic regression models [35]. Further verification of the interaction between job stress and rs1800497 genotype was conducted by SPSS macros (PROCESS) as well [36]. All of the models were adjusted for age, sex, job tenure, smoking, and alcohol consumption. A *p*-value of <0.05 was deemed to be statistically significant.

## 3. Results

### 3.1. Characteristics of the Study Population and Prevalence of Sleep Dysfunction

The characteristics of the study participants are described in Table 2. Based on the cut-off score, approximately 23.1% (160/693) of the participants were classified as having sleep dysfunction. We found group differences with respect to the prevalence of sleep dysfunction in the case of age, sex, job tenure, and smoking (*p* < 0.05). The percentages of sleep dysfunction in males and females were 19.4% and 27.2%, respectively. Participants whose job tenure was >20 years had the highest prevalence of sleep dysfunction (28.5% vs. 20.5% vs. 19.4%). The prevalence of sleep dysfunction was higher among the smoking group compared to the non-smoking group (29.0% vs. 19.2%).

### 3.2. Correlations between JS, sDA and PSQI Subscores

A partial correlation analysis was carried out to assess the correlation between JS serum DA and sleep dysfunction after adjusting for job tenure, age, sex, smoking, and alcohol consumption. The study found significant positive correlations between subjective SQ, PSD, DD and JS (*p* < 0.05), as shown in Table 3. Moreover, there were statistically significant negative correlations between the SE, DD, and serum DA levels (*p* < 0.05).

### 3.3. Association between JS and Sleep Dysfunction

Data from thorough LR analyses of the association between sleep dysfunction and JS after adjusting for job tenure, age, sex, smoking, and alcohol consumption are shown in Table 4. The score of developing sleep dysfunction was higher among those who experienced a higher level of effort-reward imbalance (OR = 2.43, 95% CI: 1.58–3.52). The significant association persisted after adjusting for all confounding factors, and greater odds of exhibiting sleep dysfunction were observed in relation to higher job effort (OR = 1.47, 95% CI: 1.01–2.12), lower job reward (OR = 2.05, 95% CI: 1.39–3.01), higher over-commitment (OR = 1.52, 95% CI: 1.05–2.21), and a higher level of effort-reward imbalance (OR = 2.13, 95% CI: 1.46-3.12).

### 3.4. Association between DRD2 Genotype and Sleep Dysfunction

Out of the 693 subjects that underwent molecular genotyping for DRD2 polymorphisms, 546 participants belonged to genotype InsIns, 139 participants with InsDel, and eight belonged to genotype DelDel for rs1799732. On the other hand, rs1800497 had 259 participants belonging to A2A2, 317 to A1A2, and the remaining 117 participants were A1A1. Using the Hardy–Weinberg equilibrium, the genotypes did not show any statistically significant differences between the normal and sleep dysfunction groups (*p* > 0.05). For rs1800497, a χ^2^ analysis revealed significant genotypic distribution differences between the two groups (*p* < 0.05). Similarly, the LR showed that the A1A1 allele (OR = 3.14, 95% CI: 1.91–5.16) increased the possibility of sleep dysfunction compared to the A2A2 allele, as shown in Table 5. The significant relationship persisted after adjusting for all other confounding factors (OR = 2.90, 95% CI: 1.75–4.82).

### 3.5. Interaction between JS, DRD2 Polymorphism, and Sleep Dysfunction

The interaction estimate between JS and DRD2 polymorphism on sleep dysfunction is shown in Table 6. Participants were divided into high or low JS groups and A1A1 or A1A2/A2A2 genotype to investigate the interaction between DRD2 polymorphism and JS concerning sleep dysfunction. We found that compared to participants with low JS levels (A1A2/A2A2 genotype), those with a high JS level (A1A1 genotype) were more likely to have sleep dysfunction (OR = 5.40, 95% CI: 2.89–10.11). Participants with high JS and the A1A2/A2A2 genotype increased the possibility of sleep dysfunction (OR = 2.07, 1.33–3.21). Interestingly, subjects with low JS and the A1A1 genotype showed an increased likelihood of sleep dysfunction (OR = 2.05, 95% CI: 1.03–4.11).

To confirm the rs1800497 × job stress interaction in sleep dysfunction, we also used SPSS macros (PROCESS), with bootstrap = 5000 and the sleep dysfunction total score as a dependent variable, job stress as an independent variable, and rs1800497 genotype as moderator. For the sleep dysfunction total score, the model summary was significant R^2^ = 0.111, F = 10.703, *p* < 0.001 and the interaction of between stress and DRD2 rs1800497 genotype was significant, with a R^2^ change of 0.007 (F = 5.661, *p* = 0.018).

## 4. Discussion

From the findings, the study observed that exposure to job stress was more likely associated with sleep dysfunction in professionals. At the molecular level, there was a significant relationship between DRD2 rs1800497 polymorphism and sleep dysfunction. There was a direct association between DRD2 rs1800497 and job stress, a relationship that increases sleep dysfunction. The study also showed that individuals with the homozygous A1A1 genotype are more susceptible to job stress than those with heterozygous alleles or homozygous A2 alleles.

### 4.1. Association between Job Stress and Sleep Dysfunction

Our study found that high job effort, low job reward, and high over-commitment were associated with sleep dysfunction. Thus, there is a close association between JS and sleep dysfunction, a finding which is largely consistent with the previous literature [13]. After considering all the relevant data, the results indicate that an ERI relates directly to sleep disturbances [12]. A prospective cohort study that was conducted on middle-aged Japanese workers found that ERI and over-commitment to work had a significant association with insomnia. The study also observed that proportionate reward for work effort helps promote recovery from insomnia [37]. Nonetheless, several studies have tried to explain this complex relationship [29].

Interestingly, we found a negative correlation between sDA level and sleep dysfunction, which was consistent with a previously reported study on DA system dysfunction [38]. Besides, the brain DA level was significantly lower in severer sleep disorders and poorer sleep quality subjects [39]. Thus, the actual relationship between sDA levels and sleep dysfunction needs further examination.

### 4.2. Association between DRD2 Gene Polymorphism and Sleep Dysfunction

Our result showed that rs1800497 homozygous A1A1 genotype increased the possibility of sleep dysfunction (OR = 3.14, 95% CI: 1.91–5.16), notwithstanding the confounding factors. TaqIA alters the expression of both DRD2 and its neighborhood gene ANNK1, thus, affecting the DRD2/ANNK1 locus [40]. Compared with the A2 allele, the DRD2 rs1800497 A1 allele was associated with a 30–40% decreased in the expression of the D2 dopamine receptor, thereby reducing the dopaminergic activity in the central nervous system [41]. This A1 allele has also been associated with reduced glucose metabolism in the striatum and the ventral and medial prefrontal cortex [42]. Moreover, the homozygous A1A1 genotype has also been linked to major depressive disorders [43] and schizophrenia [44].

DRD2 gene -141C insertion/deletion (Ins/Del) polymorphism was located in the 58th flanking region of the DRD2 gene at position −141. A meta-analysis study on the relationships between the −141C Ins/Del (rs1799732) polymorphism in DRD2 and the risk of many neuropsychiatric disorders showed that Asian Del allele carriers had decreased chances of developing many neuropsychiatric disorders, such as schizophrenia [44]. However, the protective role was not significant, probably because of the relatively small sample size used in the study.

### 4.3. The Relationship between JS and DRD2 Polymorphism on Sleep Dysfunction

The result of our study adds to the existing body of knowledge on the environmental and genetic association in sleep dysfunction. This study suggested that the relationship between high levels of JS and rs1800497 homozygous individuals (A1A1) may occur in a multiplicative fashion. In other words, despite adjusting for potential confounding factors, the interaction between the two variables remained high (OR = 5.40). Moreover, there was a significant association between genotype and sleep dysfunction among individuals who experienced low job stress, with the A1 homozygote being a major factor for sleep dysfunction in this group. The interactive relationship between the DRD2 polymorphism and job stress could be explained by a possible theory architecture: the diathesis-stress model [45]. In this model, it is proposed that individuals vary in susceptibility to adverse environmental influences, where the same negative environmental factors may have different effects or degrees of effect on different people [45]. This suggests that the DRD2 gene may be ‘plasticity genes’ for sleep dysfunction [45]. Further, evidence from neuroimaging demonstrates that the A1A1 genotype in DRD2 rs1800497 is a vulnerable gene that relates to the regulation of dopamine receptor synthesis. This pathway is essential and plays a pivotal role in the activation of the HPA axis [25,41].

### 4.4. Study Limitation

To our knowledge, this is the first study to explore the interaction between job stress and DRD2-specific SNPs on sleep dysfunction among the Chinese Han population. However, several limitations must be noted. First, we only used a cross-sectional design; thus, caution should be taken when inferring causality. The study also used a relatively small sample size (693 individuals) and might have interfered and diluted the significance of certain observations made in the study. Furthermore, DA levels were measured in serum, not in CSF. There has been no actual evidence to demonstrate that the peripheral DA levels may reflect similar CNS levels, which warrants further investigation. Moreover, the self-reporting questionnaire system used to collect sleep dysfunction data might have skewed results [46]. Fourth, other factors that may have confounded the observed association between JS and sleep dysfunction were not considered in this study [12]. Finally, we only examined two polymorphisms of two genes, which does not fully represent the underlying genetic variation. More variants of the DRD2 gene need to be examined for a more comprehensive analysis [47].

## 5. Conclusions

In summary, our results showed a remarkable relationship between the DRD2 genotype and JS concerning sleep dysfunction among the Chinese Han population. Furthermore, we found that people with both the A1 allele of the DRD2 gene and JS were more likely to have sleep dysfunction. Further prospective studies in a larger sample size from other populations and ethnic groups would help clarify the role of the DRD2 genotypes in the regulation of the association between job stress and sleep dysfunction.

## Figures and Tables

**Table 1:** Primers for amplifying DRD2 gene polymorphisms by SNaPshot.

Primer	Direction	Sequence 5′–3′
rs1799732_F	Forward	CCCCACCAAAGGAGCTGTACCT
rs1799732_R	Reverse	ATGCGGACCTCTTCCAACACCT
rs1800497_F	Forward	AAGGGCAACACAGCCATCCTC
rs1800497_R	Reverse	CACGGCCTGGCCAAGTTGTCTAA

**Table 2:** Prevalence of sleep dysfunction by subject characteristics.

Characteristics	*n*	No. Sleep Dysfunction	Prevalence	χ^2^	*p*-Value
Age					
<30	178	39	21.9%	11.979	0.007
30–40	218	36	16.5%		
40–50	196	52	26.5%		
>50	101	33	32.7%		
Sex					
Male	366	71	19.4%	5.945	0.019
Female	327	89	27.2%		
Education level					
Junior college	206	59	28.6%	5.097	0.078
Bachelor	360	75	20.8%		
Graduate & above	127	26	20.5%		
Job tenure (years)					
<10	279	54	19.4%	7.112	0.029
10–20	151	31	20.5%		
>20	263	75	28.5%		
Marital status					
Not married	123	29	23.6%	0.836	0.658
Married	532	120	22.6%		
Divorced/Widowed	38	11	28.9%		
Alcohol consumption					
Yes	371	86	23.2%	0.004	1.000
No	322	74	23.0%		
Smoking					
Yes	272	79	29.0%	8.945	0.003
No	421	81	19.2%		

**Table 3:** Correlations between job stress, serum DA, and sleep dysfunction.

Factors	Subjective Sleep Quality	Sleep Latency	Sleep Duration	Sleep Efficiency	Sleep Disturbance	Sleep Medication	Daytime Dysfunction
Job stress	0.774	0.353	0.744	0.209	0.229	0.352	0.688
*p*-value	<0.001	0.327	<0.001	0.577	0.545	0.331	0.019
Serum DA (pg/mL)	−0.326	−0.235	0.132	−0.704	−0.140	−0.335	−0.749
*p*-value	0.404	0.537	0.678	0.008	0.657	0.369	<0.001

**Table 4:** Logistic regression analysis job stress in relation to sleep dysfunction.

Variables	*n* (%)	Model 1		Model 2	
		OR (95% CI)	*p*-Value	OR (95% CI)	*p*-Value
Job effort					
Low	380 (54.8%)	1.00	—	1.00	—
High	313 (45.2%)	1.67 (1.17–2.39)	0.005	1.47 (1.01–2.12)	0.042
Job reward					
Low	365 (52.7%)	2.23 (1.54–3.24)	<0.001	2.05 (1.39–3.01)	<0.001
High	328 (47.3%)	1.00	—	1.00	—
Over-commitment					
Low	429 (61.9%)	1.00	—	1.00	—
High	264 (38.1%)	1.78 (1.24–2.54)	0.002	1.52 (1.05–2.21)	0.028
Effort-reward imbalance					
Low	349 (50.4%)	1.00	—	1.00	—
High	344 (49.6%)	2.43 (1.58–3.52)	<0.001	2.13 (1.46–3.12)	<0.001

OR, odds ratio; CI, confidence interval. Model 1 is crude. Model 2 is adjusted for age, sex, job tenure, smoking and alcohol consumption.

**Table 5:** Association between DRD2 polymorphism and sleep dysfunction.

Genotype	*n*	Sleep Dysfunction	Normal	OR (95% CI)	AOR ^a^ (95%CI)
rs1799732					
Ins/Ins	546	126	420	Reference	Reference
Ins/Del	139	33	106	1.04 (0.67–1.61)	1.03 (0.66–1.62)
Del/Del	8	1	7	0.48 (0.06–3.91)	0.38 (0.04–3.40)
*p*-value for χ^2^			0.764		
*p*-value for HWE		0.458	0.915		
rs1800497					
A2A2	259	43	216	Reference	Reference
A1A2	317	72	245	1.48 (0.97–2.25)	1.52 (0.99–2.33)
A1A1	117	45	72	3.14 (1.91–5.16) *	2.90 (1.75–4.82) *
*p*-value for χ^2^			<0.001		
*p*-value for HWE		0.207	0.848		

OR, odds ratio; CI, confidence interval; HWE, Hardy–Weinberg equilibrium; * *p* < 0.05; ^a^ Adjusted odds ratios (AOR) for age, sex, job tenure, smoking and alcohol consumption.

**Table 6:** The interaction between job stress and DRD2 polymorphism on sleep dysfunction.

Job Stress	rs1800497	Sleep Dysfunction	Normal	OR (95% CI)	AOR ^a^ (95% CI)
High	A1A1	30	30	6.49 (3.53–11.92) *	5.40 (2.89–10.11) *
High	A1A2/A2A2	76	208	2.37 (1.55–3.63) *	2.07 (1.33–3.21) *
Low	A1A1	15	42	2.32 (1.18–4.57) *	2.05 (1.03–4.11) *
Low	A1A2/A2A2	39	253	Reference	Reference

^a^ Adjusted odds ratios (AOR) for age, sex, job tenure, smoking, and alcohol consumption; * *p* < 0.05.
